# Modified Vaccinia Virus Ankara as a Viral Vector for Vaccine Candidates against Chikungunya Virus

**DOI:** 10.3390/biomedicines9091122

**Published:** 2021-08-31

**Authors:** Juan García-Arriaza, Mariano Esteban, Daniel López

**Affiliations:** 1 Department of Molecular and Cellular Biology, Centro Nacional de Biotecnología, Consejo Superior de Investigaciones Científicas (CSIC), 28049 Madrid, Spain; 2 Unidad de Presentación y Regulación Inmunes, Centro Nacional de Microbiología, Instituto de Salud Carlos III, 28220 Majadahonda, Spain

**Keywords:** poxvirus, vaccinia virus, MVA, vaccine, chikungunya virus

## Abstract

There is a need to develop a highly effective vaccine against the emerging chikungunya virus (CHIKV), a mosquito-borne *Alphavirus* that causes severe disease in humans consisting of acute febrile illness, followed by chronic debilitating polyarthralgia and polyarthritis. In this review, we provide a brief history of the development of the first poxvirus vaccines that led to smallpox eradication and its implications for further vaccine development. As an example, we summarize the development of vaccine candidates based on the modified vaccinia virus Ankara (MVA) vector expressing different CHIKV structural proteins, paying special attention to MVA-CHIKV expressing all of the CHIKV structural proteins: C, E3, E2, 6K and E1. We review the characterization of innate and adaptive immune responses induced in mice and nonhuman primates by the MVA-CHIKV vaccine candidate and examine its efficacy in animal models, with promising preclinical findings needed prior to the approval of human clinical trials.

## 1. Vaccinia Virus and the Success against Smallpox

Smallpox is a highly contagious disease that has plagued humankind as one of the most lethal pandemics, with a persistent and universal impact on the human population since its probable emergence in the first irrigated agricultural settlements. Nearly one billion deaths have been attributed to variola virus, with approximately 300–500 million deaths in the 20th century [1]. This pathogen severely altered the course of history at different times, contributing to the decline of some human civilizations, with a 30% mortality rate [2]. Early efforts to protect people against severe forms of smallpox by inoculation with smallpox scabs and/or pus (variolation) have been historically documented from medieval times in China and India. From these cultures, the variolation technique slowly extended to southwestern Asia, Japan, the Middle East and the Ottoman Empire, and arrived later to Africa, Europe and European colonies around the world [3]. Although immunization by variolation was a reasonably effective and preventive medical procedure against smallpox, some subjects inoculated with these secretions from infectious variola virus became seriously ill or died. In addition, these individuals could initiate smallpox outbreaks in susceptible populations.

After the low vulnerability of milkmaids to smallpox was observed in rural areas of European countries, Edward Jenner and other English, Dutch and German physicians inoculated different study subjects with fluid extracted from pustules on the hands and arms of cowpox- or horsepox-infected milkmaids [3,4]. This prophylactic measure was so successful that inoculation with pustule fluid quickly spread in most European countries, their overseas colonies and the newly independent United States of America (USA), starting the era of prophylactic vaccines. A remarkable historic episode in this field was the first mass vaccination campaign against smallpox, launched by Spain in 1803. In this expedition, 22 orphan boys from Galicia were utilized during the sea voyage as successive carriers of the Jenner vaccine to America. In addition, the expedition also carried scientific instruments and Spanish translations of one recent book on vaccination to be distributed to the local vaccine commissions, organized by the expedition members as they visited the different territories [5,6]. Other independent initiatives to vaccinate the American population were also successful. For example, Dr. Benjamin Waterhouse introduced the vaccine in Boston, and the vaccine was brought to California in 1817 by Russian merchants who obtained it in Peru [6].

The isolation of pustule fluids from very heterogeneous origins in different European countries and their random exchange throughout the 19th century generated complex mixtures of a vaccine [7]. The main vaccine utilized in the massive worldwide vaccination program organized by the WHO that eradicated smallpox was vaccinia virus (VACV) [8].

## 2. Second-Generation Smallpox Vaccines

In the smallpox eradication program coordinated by the WHO, different VACV strains were utilized by more than 50 different manufacturers worldwide to produce the billions of doses needed to vaccinate the world’s population. The Lister, New York City Board of Health (NYCBH), Tiantan and EM63 VACV strains were the most (but not the only ones) used in this global effort [3]. In addition, different production systems were utilized to propagate the different VACV strains, such as skin (calf, sheep, water buffalo, etc.), calf lymph or chorioallantoic membrane embryonated hens’ eggs [3].

Over time, the WHO required manufacturers to standardize the potency, safety and stability of the different vaccine batches, yielding second-generation smallpox vaccines. In this improvement process, a very important measure was the replacement of virus propagation in live animal tissues by cell culture production, a measure that greatly reduced the risk of contamination by adventitious agents [3].

## 3. Limitations of First- and Second-Generation Smallpox Vaccines

Complications arising from live VACV immunization were well documented during the vaccination program in different countries. In the USA, several adverse effects, mainly dermatologic such as eczema and vaccinia necrosum, or central nervous system disorders such as encephalitis, encephalopathy or Guillain–Barré syndrome, were seen in a thousand per million primary vaccinations [9]. In addition, in this vaccination program, a fatality rate of one in a million was also found [10]. These complications were largely associated with the relative immunosuppressive status of vaccine recipients immunized with a replication competent virus such as VACV. Cases of VACV transmission between vaccinees and close contacts were reported [4]. After the last naturally acquired case of smallpox, diagnosed in 1977, and smallpox eradication in 1979, the protection of civilian and military personnel against the deliberate dissemination of smallpox virus by terrorist action was a major consideration. Together with the emergence of the global human immunodeficiency virus (HIV) epidemic appearing in 1981 and the increasing prevalence of patients on immune-suppressive therapy, the development of safer poxvirus-based vaccines is a critical concern to protect the population against a bioterrorism attack [10].

Over the years, vaccine safety standards became more rigorous, so that at the turn of the century, the classical smallpox vaccine was contraindicated in approximately 30% of the population. This included babies, pregnant and breastfeeding women, immunocompromised patients, people with exfoliative skin disorders, eczema and cardiovascular illnesses (such as a history of angina, cardiomyopathy, congestive heart failure, myocardial infarction, stroke or transient ischemic attack). In addition, the vaccine was also contraindicated for people in close contact with individuals with the above conditions. [11]. Thus, second-generation smallpox vaccines became obsolete in practice, and efforts to develop safer vaccines against smallpox were promoted [10].

## 4. Third-Generation Smallpox Vaccines: MVA

At this point, the development of attenuated smallpox vaccines for people with contraindications to traditional smallpox vaccines was required. Among other approaches, the wild-type chorioallantois VACV Ankara (CVA) strain was serially passaged on chick embryo fibroblasts over 516 times during late 1950s and early 1960s, and renamed the modified vaccinia virus Ankara (MVA) strain [12]. In addition, during the 1970s, the process continued, and MVA reached more than 570 passages. During this long in vitro passage, six major deletions and multiple other alterations were identified in this new modified vaccinia virus Ankara (MVA) strain when compared to the genome of its parental CVA [13]. For example, 124 MVA open reading frames (ORFs) encode proteins that contain one or various amino acid exchanges or insertions/deletions compared with the gene products encoded by orthologous CVA ORFs [14]. These changes severely impede the replication of MVA in mammalian cells, but not in chick embryo fibroblasts [15]. In mammalian cells, an MVA replication blockade occurs after immature virions are formed; thus, virus-infected cells express and accumulate high levels of MVA-encoded proteins, allowing MVA to have a good immunogenicity profile and diminished virulence in mammalian hosts [16,17]. In some specialized cells, such as macrophages and dendritic cells that do not allow late gene expression, antigen generation is low [15,18]. Overall, MVA is generally referred to as a nonreplicating viral vector in human cells. Therefore, MVA is an alternative smallpox vaccine [19,20] that was used in Germany in the 1970s, close to the end of the WHO smallpox campaign [21], and was approved by the US Food and Drug Administration (FDA) on 24 September 2019, to prevent both smallpox and monkeypox. It was also approved for human use under specifications against smallpox in Europe by the European Medicines Agency (EMA) and in Canada. In addition, MVA can be lyophilized, which allows for easier and less expensive transportation, storage and distribution without cold chains, which are essential requirements in developing countries, where transport and health infrastructures are deficient. Moreover, recombinant MVA has also been used in several preclinical and human clinical trials as a vaccine candidate against numerous human infectious diseases, such as HIV/AIDS, malaria, tuberculosis, hepatitis C, emerging viruses (such as chikungunya, Zika and Ebola) and even against several tumors [22,23]. All these preclinical and clinical trials convert MVA into a reliable vaccine platform that can be used against any viral pathogen.

## 5. History, Pathology and Structure of Chikungunya Virus

Chikungunya virus (CHIKV) is an *Alphavirus* of the *Togaviridae* family that is transmitted by mosquitoes of the genus Aedes [24]. The virus causes acute febrile illness in infected people, which frequently leads to chronic debilitating polyarthritis and polyarthralgia. Most of the symptoms resolve after 10 days, but polyarthralgia can persist for months or years [25], and severe symptoms, such as encephalitis, hemorrhagic disease and mortality, have also been described [26].

This arboviral pathogen was discovered in Tanzania under British colonial administration in 1952 [27]. In the following years, several outbreaks were identified in other British colonies in Africa, probably facilitated by the political and commercial relations between the different British territories. Later, this pathogen caused frequent epidemics in Africa and Asia from the 1960s to the 1980s [28]. The relative inactivity of this virus for the following 15 years ended in 2005 with an explosive epidemic in the French overseas department of La Réunion and other Indian Ocean islands, with more than 700,000 cases and 250 deaths [29]. In 2006, several million people were affected by this pathogen in a new massive outbreak in India [30]. Since then, CHIKV has expanded rapidly to practically all tropical and subtropical regions of the world [31], with increasing severity compared with that previously reported [32]. In recent years, Italy and other European countries have also reported CHIKV outbreaks [33]. Therefore, morbidity due to this virus is a serious threat to global health, making CHIKV a high-priority emerging pathogen [34].

**Figure 1:**
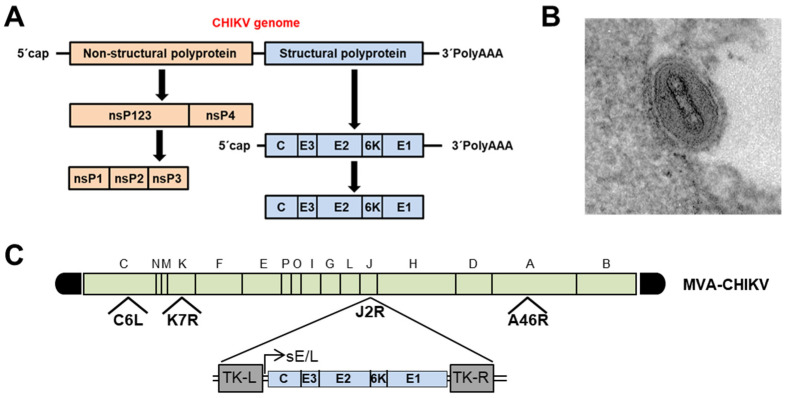
CHIKV genome organization and MVA-CHIKV vaccine candidate. (**A**) CHIKV genome. CHIKV has a single-stranded, positive-sense RNA genome of approximately 11.8 kb in length. It has 2 open reading frames (ORFs) separated by an untranslated junction region. One ORF encodes for a polyprotein, which is the precursor of the nonstructural proteins nsP1, nsP2, nsP3 and nsP4. The second ORF encodes the structural proteins capsid (**C**), envelope 1 (E1) and envelope 2 (E2) and 2 small peptides, E3 and 6K. The genome has 5′cap structures and a 3′poly A tail. (**B**) Electron microscopy image of an MVA virus particle bound to the cell membrane, produced at 12 hpi in primary chicken embryo fibroblast cells, following methods described previously (scale bar 50 nm) [36]. (**C**) MVA-CHIKV vaccine candidate. The different HindIII restriction fragments of the MVA genome are indicated by green boxes and in capital letters, as described [37], with the left and right terminal regions shown in black. Deletions of genes C6L, K7R and A46R in the MVA-CHIKV genome are also indicated. The CHIKV structural genes C, E3, E2, 6K and E1 driven by the strong synthetic early/late (sE/L) virus promoter [38] inserted within the VACV TK viral locus (J2R) are indicated [39].

The CHIKV capsid encloses an approximately 12-kb single-stranded, positive-sense RNA genome that codes for two large polyproteins [35] (Figure 1A). The first is the nonstructural P1234 precursor, which is autocatalytically processed by the C-terminal domain of nonstructural protein 2 (nsP2), releasing the four multifunctional nsP proteins. Moreover, the maturation of the structural polyprotein involves three different proteases. First, the capsid (C) is autocatalytically released, and later, two host proteases (the endoplasmic reticulum (ER) signal peptidase and furin proteases) generate the 6K transmembrane and the three E1, E2, and E3 envelope proteins [35].

## 6. Recombinant MVAs as Potential Vaccine Candidates against Chikungunya Virus

Structural CHIKV proteins are the immune system’s main target when trying to counteract CHIKV infection. Therefore, the insertion and expression of all these structural proteins in a viral vector might be a good approach to be used as a vaccine for the broad activation of B and T cell immune responses. Thus, we generated a vaccine candidate against CHIKV, termed MVA-CHIKV, based on the poxvirus MVA vector (Figure 1B). This recombinant virus expressed the CHIKV C, E3, E2, 6K and E1 structural proteins under the transcriptional control of a strong early/late VACV promoter [39] (Figure 1C).

The expression of CHIKV structural proteins was high in MVA-CHIKV-infected permissive chicken DF-1 cells and during consecutive passages, indicating that MVA-CHIKV was genetically stable. Additionally, MVA-CHIKV showed similar kinetics of viral growth in permissive chicken DF-1 cells to parental MVA, indicating that the constitutive expression of the five CHIKV structural proteins does not impair vector replication under permissive conditions [39]. Moreover, real-time PCR analyses showed that MVA-CHIKV triggers a strong innate immune response in human monocyte-derived dendritic cells and macrophages, with the expression of several chemokines, such as MIP-1α, IP-10, and RANTES, proinflammatory cytokines, such as TNF-α and interferon (IFN)-β, and IFN-inducible genes, such as IFIT1 and IFIT2, and other key cytosolic sensors that lead to antiviral IFN production, such as RIG-I and MDA-5 [39].

In the C57BL/6 mouse model, the intraperitoneal single-dose administration of 1 × 10^7^ PFU of the MVA-CHIKV vaccine candidate induced strong, broad and polyfunctional adaptive CHIKV-specific CD8^+^ T cell immune responses. These activated cells produced IFN-γ, TNF-α and IL-2 cytokines, as well as the expression of the indirect marker of cytotoxicity CD107a against multiple peptides from CHIKV C, E1 and E2 proteins [39]. Additionally, the MVA-CHIKV vaccine candidate activated different T memory subpopulations in immunized animals, such as T effector memory, T effector cells and abundant T central memory [39]. Furthermore, a single dose of MVA-CHIKV elicited strong humoral immune responses against CHIKV with high titers of neutralizing IgG antibodies, which were further enhanced by the second immunization with the MVA-CHIKV vaccine candidate [39]. The induction of both humoral and cellular adaptive immune responses with a single immunization with the MVA-CHIKV vaccine candidate protected mice against a challenge with a high dose of CHIKV administered in the footpad. MVA-CHIKV-vaccinated mice did not develop footpad swelling and showed no virus in their blood after the challenge [39]. In contrast, severe footpad swelling and high virus titers of CHIKV in the blood were detected in parental MVA-immunized mice [39].

To identify natural CHIKV ligands presented by human leukocyte antigen (HLA) molecules, the infection of large amounts (approximately 4 × 10^10^) of human cells with a replication competent recombinant VACV (Western Reserve strain) expressing the same CHIKV structural proteins as the MVA-CHIKV vaccine candidate was carried out. This was followed up by high-throughput mass spectrometry analysis of complex HLA-bound peptide pools. Eighteen different viral ligands from the CHIKV structural polyprotein naturally presented by diverse HLA-A, -B, and -C class I and HLA-DR and -DP class II molecules were identified [40]. In addition, different epitopes generated in the cellular immune response against the CHIKV structural polyprotein were identified using transgenic mice expressing different HLA class I and II molecules, providing an in-depth characterization of the repertoire of HLA-restricted epitopes formed during the infection of human cells with a vaccine vector [40,41].

The MVA-CHIKV vaccine candidate also induced strong VACV-specific humoral and cellular immune responses [39]. Thus, the use of different vectors encoding a shared immunogen in heterologous prime-boost regimens is common to avoid buildup of antivector immunity and to increase immunogenicity against the selected pathogen [42]. In this context, priming with different construct vaccines that expressed some proteins of CHIKV, followed by heterologous booster immunizations with the MVA-CHIKV vaccine candidate, improved the CHIKV-specific immune responses with higher T cell numbers and stronger antibody titers than mice inoculated once with infectious CHIKV targeted against multiple T and B cell epitopes [43].

Prior to the study of MVA-CHIKV in humans, different regimens with this vaccine candidate and two other CHIKV vaccine candidates were also evaluated in nonhuman primates [44]. These other two vaccine candidates were an attenuated CHIKV with a deletion of 180 nucleotides of the nsP3 replicase gene region [45] and a DNA replicon (DREP) vaccine candidate derived from a CHIKV-infectious cDNA clone from which the gene region encoding the CHIKV C protein was deleted [46]; thus, this construct only expresses the CHIKV envelope proteins (E3-E2-6K-E1). As in mice, antibody levels in macaques were further improved with a second immunization, and heterologous immunization consisting of priming with the DREP vaccine candidate followed by MVA-CHIKV as a boost was the most potent regimen in the induction of antibodies and T cell responses against CHIKV C, E1, and E2 proteins [44]. All animals immunized with this prime-boost vaccine regimen and challenged with CHIKV showed neither CHIKV in their blood nor any sign of fever at any time, achieving sterile immunity [44]. The analysis of the cytokine profiles of vaccinated macaques showed no expression of cytokines typically upregulated by pathogenic CHIKV, such as IFN-α2, IL-1Ra, IL-6, IL-15 and MCP-1, and the upregulation of other cytokines, such as IL-2 and IL-4, typically associated with antiviral and memory immune responses [44].

MVA itself represents a reliable vaccine platform [18,22,47]. In addition, the results obtained in mice [39,43,46] and nonhuman primates [44] suggest that priming with the DREP vaccine candidate followed by MVA-CHIKV as a boost is a promising combined CHIKV vaccination strategy. These promising results should be studied in depth in future human clinical trials.

MVA-CHIKV expressing only E3 and E2 structural proteins generated low levels of neutralizing antibodies [48,49]. In contrast, MVA-CHIKV, including E3, E2, 6K and E1 structural proteins, generated high levels of neutralizing antibodies [39,49]. This difference seems to be because, in the absence of E1 protein, the E2 protein is improperly folded and not transported efficiently to the plasma membrane, and thus, a defective humoral response is generated.

In addition to the MVA-CHIKV expressing the CHIKV C, E3, E2, 6K and E1 structural proteins described above, other recombinant MVAs have been generated as CHIKV vaccine candidates. A summary of the characteristics of the different MVA-based vaccine candidates against CHIKV is included in Table 1. First, Weger-Lucarelli et al. inserted the CHIKV E2 and E3 genes into a cDNA clone that contained the coral Discosoma sp. red fluorescent (DsRed) gene to generate a recombinant MVA expressing the DsRed protein to allow rapid visual-based selection (MVA-CHIK) [48]. This vaccine candidate was tested in BALB/c immunocompetent mice as well as in α/β interferon signaling-deficient A129 mice, showing complete protection from viremia and mortality upon challenge with CHIKV after two doses. Conspicuously, the humoral response generated in both mouse models was characterized by high anti-CHIKV virus antibodies. However, low or undetectable levels of neutralizing antibodies were produced in these animals [48]. In contrast to natural infection, where the expression of E2 protein was observed in the supernatant of CHIKV-infected cells, no extracellular detection of E2 expression with the MVA CHIK was found, indicating that the viral envelope protein was maintained inside the cell and did not reach the cell surface in the MVA-CHIK expressing E2 and E3 proteins [48]. This fact suggests that the protective immune humoral response is severely impaired in the absence of the complete CHIKV envelope protein. In addition, the passive transfer of MVA-CHIK immune serum from immunized mice to naïve animals did not protect against CHIKV mortality, suggesting that the antibodies elicited with this recombinant vaccine are not the main effectors of protection [48]. Subsequent experiments showed that the depletion of CD4^+^, but not CD8^+^ T cells produced the death of all vaccinated animals, evidencing the crucial role of CD4^+^ T cells in the protection by MVA-CHIKV-expressing E2 and E3 proteins [48]. Thus, recombinant MVA-CHIK appears to provide protection by a CD4^+^-T-cell-dependent mechanism independent of CD8^+^ T cells and serum circulating neutralizing antibodies.

Moreover, van den Doel et al. generated three recombinant MVAs expressing different constructs of envelope polyproteins, namely, E3 and E2, 6K and E1, and the E3, E2, 6K and E1 proteins MVA-E3E2, MVA-6KE1 and MVA-E3E26KE1, respectively [49]. After single-dose immunization with MVA-E3E2 or MVA-6KE1, α/β interferon signaling-deficient A129 mice generated low levels of neutralizing antibodies [49]. In contrast, a single dose of recombinant MVA-E3E26KE1 induced significantly higher levels of neutralizing antibodies in the same immunodeficient mouse model compared to MVA-E3E2 or MVA-6KE1 vaccine candidates [49]. In the groups of animals vaccinated with MVA-6KE1, partial protection against CHIKV lethal infection was found. All mice immunized with MVA-E3E2 and MVA-E3E26KE1 were protected against CHIKV challenge [49]. CHIKV RNA was found in the liver, spleen and brain of most mice immunized with MVA-E3E2 or MVA-6KE1 after CHIKV challenge. Very low levels of CHIKV RNA were detected in the spleen and liver, but not in the brain, of animals that received the MVA-E3E26KE1 vaccine candidate and were challenged with CHIKV [49]. In addition, low levels of infectious CHIKV were isolated from the spleen of all mice immunized with MVA-6KE1 but not with MVA-E3E2 or MVA-E3E26KE1 vaccine candidates [49]. Finally, second booster vaccination improved the neutralizing antibody titers with the three recombinant MVAs, but the antibody response was significantly higher in the animals vaccinated with the MVA-6KE1 construct compared to the other two groups, a surprising result that needs further study [49], suggesting that the 6KE1 construct may be a candidate vaccine itself.

**Figure 2:**
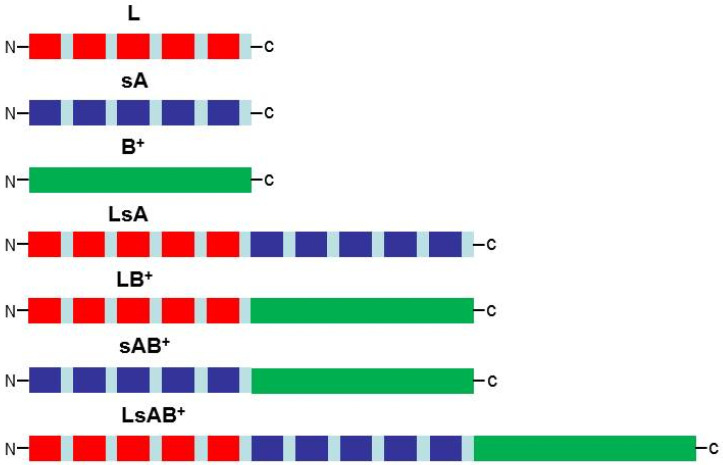
Schematic representation of the different combinations of CHIKV E2 protein fragments. Red: neutralizing epitope; dark blue: surface exposed domains of A; green: domain B with β-ribbon connector; light blue: G-S linker.

Finally, a comparative analysis of immunogenicity was performed with purified protein components and an MVA vector. Weber et al. analyzed in depth the humoral and protective immune responses against CHIKV, generating seven different recombinant E2 proteins [50] (Figure 2). First, a DNA construct including five putatively surface-exposed linear antigens of domain A from the CHIKV E2 protein assembled with glycine-serine (G-S) linkers was named sA. Five repeats of one of the putative linear antigens included in the sA construct assembled with G-S linkers were included in the recombinant L. Additionally, a recombinant protein expressing both L and sA constructs in tandem was generated. In addition, the whole domain B from the CHIKV E2 protein, including a fragment of the surface-exposed β-ribbon connector, was used to produce recombinant B^+^. Similar to LsA, recombinants LB^+^ and sAB^+^ were also generated. Finally, a recombinant protein including the three constructs L, sA, and B^+^ was also generated [50]. Immunocompetent mice were immunized with three doses of each of the seven recombinant proteins purified by Ni^2+^ affinity chromatography from *E*. *coli* plus adjuvant. Animals immunized with the recombinant proteins L and sA did not induce neutralizing antibodies. The significant induction of specific neutralizing antibodies was detected in the sera of animals immunized with the other five recombinant proteins. B^+^-, LB^+^- and especially sAB^+^-immunized mice induced the highest amount of neutralizing antibodies. Thus, domain B^+^, alone or in combination with others (including sA that did not generate a remarkable response), was sufficient to induce neutralizing antibodies in animals [50]. Four immunizations with sAB^+^ protein or with MVA-CHIK-sAB^+^ or the mixture of both immunogens were tested in immunocompetent mice against a challenge with infectious CHIKV. Recombinant sAB^+^ protein decreased the CHIKV titer in serum at day 2 but not at day 4. Conversely, MVA-CHIKV-sAB^+^ reduced the CHIKV titer in serum at day 4 but not at day 2. In addition, decreased viral titers in the spleen, but not in the lungs, with recombinant protein immunizations were found. However, MVA-CHIKV-sAB^+^ reduced the viral titer in the lungs but not in the spleen [50]. These results indicated partial protection against CHIKV infection using putative surface-exposed, linear antigens of domain A and the whole domain B from CHIKV E2 protein as recombinant protein or MVA vector; this protection was not increased when the mixture of these two immunogens was administered to the mice [50].

## 7. Other Vaccination Strategies against CHIKV

In addition to recombinant MVA, other potential vaccine candidates against CHIKV are currently in development using different strategies (reviewed in [51]). Inactivated vaccines against CHIKV have been developed since the 1970s. In general, they are stable and safe, stimulating both cellular and humoral immune responses. However, their production costs prevent accessibility in practice. Insect cells or baculovirus expression systems have been utilized to generate CHIKV envelope glycoprotein E1 and E2 subunit vaccines that elicit immune responses without the production of Abs against unrelated antigens or anti-vector immunity. Subunit vaccines are safe and make large-scale manufacturing possible. In contrast, the titers of neutralizing Abs obtained and mouse protection against challenge were moderated compared with other vaccination strategies. The generation of live attenuated viruses significantly decreases their virulence while preserving their immunogenicity. However, higher immunogenic ability is a tradeoff for lower safety. Several substitutions, deletions and stop codons in different CHIKV nonstructural proteins and capsids are currently being evaluated. In addition to MVA described in this review, other recombinant virus vectors are being studied in CHIKV vaccine development. All of them offer the advantages of safety and easy inoculation. Recombinant measles-virus-encoding CHIKV structural proteins generated a vigorous immune response in macaques. This vaccine also showed effectiveness in both phase I and II clinical trials. Different CHIKV structural proteins inserted into the adenovirus genome induced high neutralizing IgG titers against CHIKV. Additionally, recombinant VSV with CHIKV structural proteins induced neutralizing Abs in mice, with no viremia detectable. The generation of CHIKV virus-like particles assembled in yeast elicited a significant humoral and cellular immune response and good protection in mice. Additionally, a chimeric vaccine from Eilat virus, an insect-specific *alphavirus*, combines the deficiency of replication in human cells with the entry, delivery of RNA and synthesis of CHIKV structural proteins; in mice and macaques, it showed the induction of humoral and cellular immune responses as well as protection against CHIKV challenge. Finally, a DNA vaccine encoding the full-length infectious genome of a live attenuated CHIKV clone generated neutralizing Abs in mice, but low frequencies of reversion were identified.

## 8. Concluding Remarks

Morbidity due to CHIKV is a serious threat to global human health and is currently considered a high-priority emerging pathogen. Therefore, a protective vaccine that specifically promotes an antiviral immune response against this virus must be developed. Various investigations have been directed toward developing an effective vaccine against CHIKV infection using different technologies, including live-attenuated CHIKV, nonrelated recombinant viral vectors, replicons, protein components and recombinant DNA and mRNA platforms encoding different viral proteins. In mice and monkeys immunized with those vaccine candidates, differential degrees of immunogenicity and efficacy were observed, with some reaching sterile immunity [39,44,52,53]. The main protein targets identified were the structural CHIKV proteins E3, E2, 6K and E1, as these proteins elicit neutralizing antibodies and T cell responses, considering that both arms of the immune system are needed in the control of viral infection. Each delivery platform has its own advantages and disadvantages. In the case of attenuated live viral vectors, these vectors tend to produce wider immune responses than purified protein components or inactivated virus, with the disadvantage that immune responses are directed against the vector protein components as well as against the foreign antigen; hence, repeated immunizations with the same live attenuated vector might diminish the booster effect. This can be circumvented by combined vaccination with two different vectors [22].

In the design of the MVA-CHIKV, the consideration was to include all of the viral genes encoding the C, E3, E2, 6K and E1 structural proteins to generate a vector triggering broad immune responses. This candidate vaccine induces strong, broad and polyfunctional adaptive CHIKV-specific CD8^+^ T cell and humoral immune responses in mice that fully protected the animals against a challenge with infectious CHIKV. In addition, in nonhuman primates, the MVA-CHIKV vaccine candidate elicited sterile immunity, with no virus in blood and no fever after CHIKV challenge when combined with a DREP vaccine candidate, and protection correlated with strong humoral and cellular immune responses.

Remarkably, there are several advantages of the recombinant MVA vectors over other live attenuated vectors for vaccine usage: (i) the high safety of the vector in humans; (ii) the acceptability of large inserts within the viral DNA genome; (iii) the high expression of heterologous gene(s); (iv) the potent capacity to induce a broad spectrum of B and T cell immune responses; (v) the durability of these immune responses; (vi) the high stability of the inserts in the viral genome; (vii) the high stability of the vaccine at room temperature, under refrigerator conditions, as well as a lyophilized product; and (viii) reduced cost. These characteristics make MVA vectors promising vaccine candidates that merit further exploration in human clinical trials.

To date, the only licensed vaccine based on a MVA vector is MVA-BN-Filo, which is used in the prime/boost protocol against Ebola together with an adenovirus vector [23]. A problem derived from the use of MVA against pathogens could be a decreased immune response in individuals with previous immunization to other recombinant MVA or exposure to other natural orthopoxviruses. However, it has been observed that individuals previously vaccinated against smallpox or participating in clinical trials with MVA vectors showed good responses during repeated immunization [20]. In addition, the use of a poxvirus vector as part of a heterologous prime/boost combination of vectors has shown excellent immune responses in different animal model systems, which reduces the problem of immune interference [47].

For MVA vectors to advance further into clinical application, there is the need to use them in a more widely distributed human disease. With the SARS-CoV-2/COVID-19 pandemic affecting the world population and, to fight the disease, many different vaccine strategies have been developed, and several of these vaccines have shown high efficacy in humans [54]. One type of vaccine candidate is based on recombinant MVA, which has shown good immunogenicity profiles and high efficacy against SARS-CoV-2 in preclinical trials [55,56,57,58,59], supporting further development in clinical trials. It remains to be defined how far MVA-expressing SARS-CoV-2 antigens will move forward against COVID-19 (ClinicalTrials.gov), but it will be a good opportunity to evaluate the effectiveness and long-term durability, a feature of smallpox vaccination [1]. Much still remains to be learned on correlates of protection and the efficacy roles of B and T cell responses of CHIKV-based vaccines from preclinical to clinical trials. Additionally, how these vaccines protect against the pathological changes produced by CHIKV infection should be investigated under conditions where the disease is prevalent.

In summary, there are several MVA-based vaccine candidates that have been shown to be effective in preclinical studies and should be considered for further development of a vaccine against CHIKV to avoid a potential situation like the one we are currently experiencing with the COVID-19 pandemic.

## Figures and Tables

**Table 1:** Summary of recombinant MVA vaccine candidates against CHIKV.

Name	CHIKV Viral Proteins Expressed	Vaccination Strategy ^a^	In Vitro Effects	In Vivo Effects (Mice or Nonhuman Primates)	Limitations	Reference
MVA-CHIKV (C/E3/E2/6K/E1)	C, E3, E2, 6K and E1 structural proteins	Single and/or double dose	Multiprotein expression. Activation of human dendritic cells and macrophages.	- Activation of CD8^+^ T cells. - High levels of neutralizing IgG Abs. - 100% protection of mice against CHIKV challenge. - Production of cytokines and neutralizing IgG Abs in macaques. - 100% efficacy in macaques when combined in prime/boost with an *alphavirus* replicon.		[39,40,41,43,44]
MVA-CHIK	E3 and E2 structural proteins	Two doses	E3/E2 expression	- High anti-CHIKV virus Abs in mice. - Protection of mice against CHIKV challenge.	Low or undetectable levels of neutralizing Abs.	[45]
MVA-E3E2 (E3/E2)	E3 and E2, structural proteins	Single dose	E3/E2 expression	- Low levels of neutralizing Abs in mice. - Protection of mice against CHIKV challenge.	Low levels of neutralizing antibodies.	[46]
MVA-6KE1 (6K/E1)	6K and E1 structural proteins	Single dose	E1 expression	- Low levels of neutralizing Abs in mice. - Partial protection against CHIKV lethal infection in mice.	Low levels of infectious CHIKV were isolated from the spleen of mice immunized.	[46]
MVA-E3E26KE1 (E3/E2/6K/E1)	E3, E2, 6K and E1 structural proteins	Single dose	E3/E2/E1 Expression	- High levels of neutralizing Abs in mice. - Protection of mice against CHIKV challenge.		[46]
MVA-CHIKV-sAB^+^	5 putative linear antigens of domain A from CHIKV E2 protein assembled with glycine-serine plus the whole domain B from E2	Four doses	Multiepitope expression	- Reduction in CHIKV titer in serum at day 4. - Reduction in viral titer in lungs.	- No reduction in CHIKV titer in serum at day 2. - No reduction in viral titer in spleen.	[48]

^a^ Recombinant MVA vaccine candidates are all low cost and present high stability.

## Data Availability

Not applicable.
